# Release and Constancy of an Antibiotic Resistance Gene in Seawater under Grazing Stress by Ciliates and Heterotrophic Nanoflagellates

**DOI:** 10.1264/jsme2.ME17042

**Published:** 2017-06-08

**Authors:** Thi Lan Thanh Bien, Ngo Vy Thao, Shin-Ichi Kitamura, Yumiko Obayashi, Satoru Suzuki

**Affiliations:** 1 Center for Marine Environmental Studies, Ehime UniversityMatsuyama, EhimeJapan; 2 The United Graduate School of Agricultural Sciences, Ehime UniversityMatsuyama, EhimeJapan; 3 Department of Biotechnology, Nong Lam UniversityHo Chi Minh CityVietnam; 4 Faculty of Environment and Resources, Nong Lam UniversityHo Chi Minh CityVietnam

**Keywords:** antibiotic resistance gene, grazing, ciliate, heterotrophic nanoflagellate

## Abstract

Extracellular DNA (exDNA) is released from bacterial cells through various processes. The antibiotic resistance genes (ARGs) coded on exDNA may be horizontally transferred among bacterial communities by natural transformation. We quantitated the released/leaked tetracycline resistance gene, *tet*(M) over time under grazing stress by ciliates and heterotrophic nanoflagellates (HNFs), and found that extracellular *tet*(M) (ex-tetM) increased with bacterial grazing. Separate microcosms containing *tet*(M)-possessing bacteria with ciliates or HNFs were prepared. The copy number of ex-tetM in seawater in the ciliate microcosm rapidly increased until 3 d after the incubation, whereas that in the HNF microcosm showed a slower increase until 20 d. The copy number of ex-tetM was stable in both cases throughout the incubation period, suggesting that extracellular ARGs are preserved in the environment, even in the presence of grazers. Additionally, ARGs in bacterial cells were constant in the presence of grazers. These results suggest that ARGs are not rapidly extinguished in a marine environment under grazing stress.

Antibiotic resistance is a serious and growing public health issue worldwide (17). Antibiotic resistance genes (ARGs) are found in natural aquatic environments as intracellular and extracellular ARGs (54). ARGs in bacterial cells may spread by conjugation and transduction among bacterial communities (29). ARGs coded on extracellular DNA (exDNA) are released from live antibiotic-resistant bacteria (ARB) (41) or decayed ARB (51) through, for example, grazing and virus infection (1, 31). These ARGs on exDNA may represent a source for natural transformation (27, 30, 31), which may also drive the evolution of ARGs in natural microbial communities (5, 37). Previous studies demonstrated that exDNA was not decomposed over time in various environments (30, 34), and ARGs also remained in the environment. However, a quantitative study has not yet been conducted on the production and degradation of ARGs in a seawater environment.

The interaction between different microbes has been reported to release exDNA into environments (31). Ciliates and heterotrophic nanoflagellates (HNFs) are important members of the microbial loop (2, 40), functioning as common grazers and controlling the population of marine bacteria (1, 3, 13). These protists also transport organic matter to higher trophic levels (56) as well as nutrient remineralizers (32). Ishii *et al.* (18) demonstrated that grazing by ciliates in a freshwater hypereutrophic pond was an important source of bacterial exDNA. The production of exDNA was also observed in microcosms containing *Escherichia coli* and the ciliate *Tetrahymena thermophila* (21), and the co-cultivation of *E. coli* with algae or cyanobacteria also increased extracellular plasmid DNA (31).

It currently remains unclear whether ARGs coded on exDNA persist in aquatic environments. There is no conclusive evidence on the effects of grazing on the degradation of ARGs in a marine environment. exDNA may be less stable because it is easily damaged by physical and chemical factors as well as nucleases (34). On the other hand, Kaneko and Itaya (20) demonstrated that the plasmid released from lysed *E. coli* was stable and then taken up by co-existing competent *Bacillus subtilis* cells. The latter finding suggests that if ARGs are stable in seawater after grazing, they may form a pool of transferable ARGs in a marine environment.

*Photobacterium damselae* subsp. *damselae* (formerly *Vibrio damselae*) is a marine bacterium of unknown abundance that may cause infections in fish and humans (42). The marine origin strain 04Ya311 harbors a novel multi-drug resistance-transferable plasmid (pAQU1) that may be transferred to *E. coli* by transconjugation (33, 35). These characteristics suggest that the 04Ya311 strain is a promising model for multi-drug resistance in marine bacteria. Our recent findings demonstrated that pAQU1 may be retained in various bacterial hosts under oligotrophic conditions without selection pressure (4). Cairns *et al.* (6) indicated that grazing increases the persistence and spread of a resistance conjugative plasmid in pathogenic bacteria.

The present study aimed to clarify the involvement of marine ciliates and HNFs in the release/leakage of ARGs on multi-drug resistance plasmids from bacteria in seawater. The persistence of ARGs in bacterial cells under grazing pressure was also examined.

## Materials and Methods

### Bacteria and protist strains

The bacterium strain *Photobacterium damselae* subsp. *damselae* 04Ya311, which harbors the multi-drug resistance conjugative plasmid pAQU1 was isolated from seawater from the Seto Inland Sea (35). This bacterium cannot grow in medium without salt, and the optimum salt (NaCl) concentration is 2–3.5%. The entire sequence of pAQU1 has already been reported (35, 36). Strain 04Ya311 was grown in Mueller-Hinton Broth (MHB; Becton, Dickinson and Company, Franklin Lakes, USA) supplemented with 2% NaCl and 60 μg mL^−1^ oxytetracycline (OTC; Nacalai Tesque, Kyoto, Japan).

As grazer species, we used the clonal ciliates *Paranophrys marina* (46) and *Parauronema virginianum* isolated from the coastal sea of Ehime Prefecture, Japan. These ciliates feed on bacteria through a buccal apparatus. The HNF strains *Paraphysomonas imperforata* (IOW15) and *Cafeteria roenbergensis* (IOW23) were isolated by Weber *et al.* (55) and generously gifted by Professor Ramon Massana from the Institut de Ciències del Mar, CSIC, Spain. By using four species of ciliates and HNFs, a general conclusion may be provided for ciliates and HNFs rather than species-specific phenomena. *P. marina*, a free-living and parasitic ciliate, was described by Thompson and Berger (49). Its cell size *in vivo* is approximately 30–45×10–15 μm. This ciliate has been tentatively assigned to the family *Uronematidae* (47), which also comprises the genera *Uronema* and *Miamiensis* (48). The abundance and ecological importance of *P. marina* in the sea currently remain unclear (46). The genus *Parauronema* was first reported by Thompson (50) and includes three species: *P. virginianum* Thompson 1967, *P. acutum* Small and Lynn 1985, and *P. longum* Song 1995. *P. virginianum*, a member of the subclass *Scuticociliatia*, is commonly found worldwide and exhibits great biological and morphological diversity (39). This strain has a very similar morphology to *Uronema marinum* with a cell size of approximately 30–40×25–35 μm (38). Corliss (8) placed *Parauronema* in the family *Philasteridae*. Lynn (28) subsequently placed it in the family *Parauronematidae* with *Glauconema*, *Miamiensis*, and *Potomacus*. However, according to Gao *et al.* (15), *Parauronema* is phylogenetically distant from the family *Philasteridae* and, thus, from *Miamiensis*. Most ciliates feed on particulate matter consisting mainly of microorganisms (bacteria and algae) of a size appropriate to their buccal apparatus (8): suspended or settled particles are collected via specialized cilia near the oral opening. A previous study reported that the co-existence of ciliates with bacteria results in the release of proteases into the surrounding environment, which may play a role in protein digestion outside cells (46). *P. imperforata*, a heterotrophic and free-living flagellate within the class *Chrysophyceae*, is found in several coastal environments in very low abundances (typically <50 cells mL^−1^). Its cells are typically spherical and range between 4 and 8 μm in diameter (26). This species feeds by the direct interception of suspended prey including bacteria, microalgae, and other protists (11). *C. roenbergensis*, a small bacterivorous marine flagellate, was discovered by Fenchel and Patterson (14) and is typically found at depths of 52–55 m. Cells are D-shaped, 2–5 μm long, and laterally compressed, with a shallow groove on the left side of the cell. *C. roenbergensis* occurs in all oceans, and may grow to a very high concentration (in excess of 10^4^ cells mL^−1^). This species resembles *C. minuta*, *C. marsupialis*, and *Aeronema sippewissettensis* (25). *P. imperforata* and *C. roenbergensis* are naturally dominant members of the protist community in the brackish Baltic Sea (55). The major feeding mechanism of HNFs is size-selective, which shifts bacterial communities toward smaller cell sizes (16). The grazing of flagellates on bacteria was demonstrated to release phosphorus and nitrogen into the surrounding environment, which may be taken up by other organisms (12).

Ciliates and HNFs were pre-cultured at 20°C for one week in seawater that was filtered (pore size, 0.45 μm) and autoclaved, and supplemented with heat-killed *E. coli* cells as a food source.

### Microcosm preparation

The terms co-culture and mono-culture are defined as a microcosm with more than two species and that with one species, respectively. Microcosm experiments were performed in duplicate in 500-mL narrow-mouth polycarbonate square bottles (Thermo Fisher Nalgene^®^), which contained 250 mL of filtered-autoclaved seawater collected from coastal seawater off Matsuyama, Ehime Prefecture, Japan (33°54′13.24″ N, 132°42′34.05″ E) in July 2015. Seawater parameters were as follows: water temperature 20.7°C, pH 8.7, and salinity 30 (psu). An overnight bacterial culture was harvested by centrifugation, and cells were then washed twice and resuspended in filtered-autoclaved seawater. Bacterial cells (04Ya311 strain, 10^7^–10^8^ CFU mL^−1^) and protist cells (10^3^–10^4^ cells mL^−1^) were inoculated into microcosms. The initial community composition on d 0 in each microcosm is shown in Table 1. Microcosms with only bacteria or protists were used as control mono-cultures. Bottles were gently inverted once a d. Subsamples (25 mL) were withdrawn on d 0, 3, 7, 10, and 20. Ten out of 25 mL was used for the cell counting of bacteria and protists, while the remaining 15 mL was used for DNA extraction from extracellular and cellular fractions. In order to examine the effects of a mixture of three organisms (a ciliate, HNF, and bacterium), a co-culture microcosm containing *P. marina*, IOW15, and 04Ya311 was prepared. A microcosm without 04Ya311 was also prepared as a control. All microcosms were incubated at 20°C for 20 d in the dark.

### Cell enumeration

The colony-forming unit (CFU) of 04Ya311 was counted on an MHB plate containing 2% NaCl and 60 μg mL^−1^ sulfamethoxazole (SMX) after an incubation at 25°C overnight. SMX was used in this experiment to inhibit the growth of ciliate-associated bacteria. Ciliates and HNFs were directly counted according to a previous study (46). In brief, ciliate and HNF cells were fixed with glutaraldehyde (final concentration, 2% [v/v]) and incubated at 4°C overnight. Fixed samples were double stained with 4′,6-diamidino-2-phenylindole (DAPI, final concentration, 1 μg mL^−1^) and fluorescein iso-thiocyanate (FITC, final concentration, 0.5% [v/v]) for 10 min in the dark. Cells were collected on 0.8-μm black nucleopore membranes (Whatman), washed several times with ice-cold 0.5 M sodium carbonate buffer (pH 9.5), and then counted using an Olympus BX51 fluorescence microscope (Olympus Optical, Tokyo, Japan).

### DNA extraction

Each subsample (15 mL) was filtered through 47-mm polycarbonate membranes with a pore size of 0.22 μm (Millipore, USA) to collect bacteria and protists cells. All cells on filters were processed as described by Dempster *et al.* (10) to extract intracellular DNA. Total exDNA from the filtrate was precipitated by adding sodium acetate (final concentration, 0.3 M) and ethanol (final concentration, 70%) (44), and resuspended in TE buffer. exDNA was purified using a previously described method (7). In brief, exDNA was precipitated by adding 1 volume of a cetyltrimethylammonium bromide (CTAB) solution (1% CTAB in 50 mM Tris-HCl, 10 mM EDTA, pH 8.0), followed by an incubation at 65°C for 30 min and then centrifugation at 5,000×*g* at 4°C for 10 min. The pellet was resuspended in high-salt TE buffer (10 mM Tris-HCl, 0.1 mM EDTA, 1 M NaCl, pH 8.0). A 0.6 volume of cold isopropanol was then added, followed by an incubation for 1 h on ice and then centrifugation at 10,000×*g* at 4°C for 15 min. The pellet was resuspended in TE buffer, an equal volume of phenol-chloroform-isoamyl alcohol (25:24:1 [v/v/v]) was added, and the suspension was then centrifuged at 10,000×*g* for 5 min. Each supernatant was mixed with an equal volume of chloroform-isoamyl alcohol (24:1 [v/v]) and centrifuged again. DNA in the supernatant was then precipitated with sodium acetate and cold ethanol (final concentration, 70%) at −20°C for 1 h, and centrifuged at 10,000×*g* for 15 min. The pellet was then washed twice with 70% ethanol, dried, and resuspended in TE buffer.

### Gene quantitation

The pAQU1 plasmid codes seven ARGs (35), among which the tetracycline resistance gene, *tet*(M), was targeted to measure the copy number of extracellular and intracellular pAQU1. In the case of extracellular *tet*(M) (ex-tetM), the positive sign of *tet*(M) may not be derived from the entire plasmid pAQU1 because the plasmid carrying *tet*(M) may be degraded into fragments. Large extracellular plasmids are reported to be fragile and readily sheared into small pieces or digested by nucleases (9, 19). Thus, we used the term “ex-tetM copy number” instead of “pAQU1 copy number” for extracellular fractions. The copy number of intracellular *tet*(M) was normalized using *hlyA*, which is positive in 04Ya311, but not in protist-associated bacteria. Primer sets have previously been reported by Tamminen *et al.* (45) for *tet*(M) (forward-5′-GCAATTCTACTGATTTCTGC-3′, reverse-5′-CTGTTTGATTACAATTTCCGC-3′, amplicon size 186 bp), and by Rivas *et al.* (43) for *hlyA* (forward-5′-AATGTTTCTTTCCGTTGGGC-3′, reverse-5′-CCGGAGTTCCACCAGTAAAT-3′, amplicon size 353 bp). Quantitative real-time PCR (qPCR) was performed using the CFX96 Real-time System (Bio-Rad Laboratories, Hercules, CA, USA). The qPCR program for the *tet*(M) gene was described previously (4), while that for *hlyA* was at 95°C for 30 s, 40 cycles at 95°C for 10 s, and 57°C for 30 s. The following plasmids were used as controls: pGEM-*tet*M (23) and pGEM-*hlyA*, which has the insertion of the *hlyA* gene amplified with a primer set (forward-5′-ATGGTTGATGGGTCAAAAAC-3′, reverse-5′-GGTCTTAGTAGCTTATCTGC-3′, amplicon size 1,766 bp).

### Data analysis

Experiments for each protist species were performed in duplicate. Results are indicated as the average of two independent experiments. In statistical analyses, data from two species of ciliates or HNFs were combined (as *n*=4) and the significance of differences was tested by a one-tailed Student’s *t*-test to compare pairs of time points using Excel software. Each qPCR value was the average of triplicate measurements with standard errors.

## Results

### Changes in bacteria and protist abundances

**Fig. 1:**
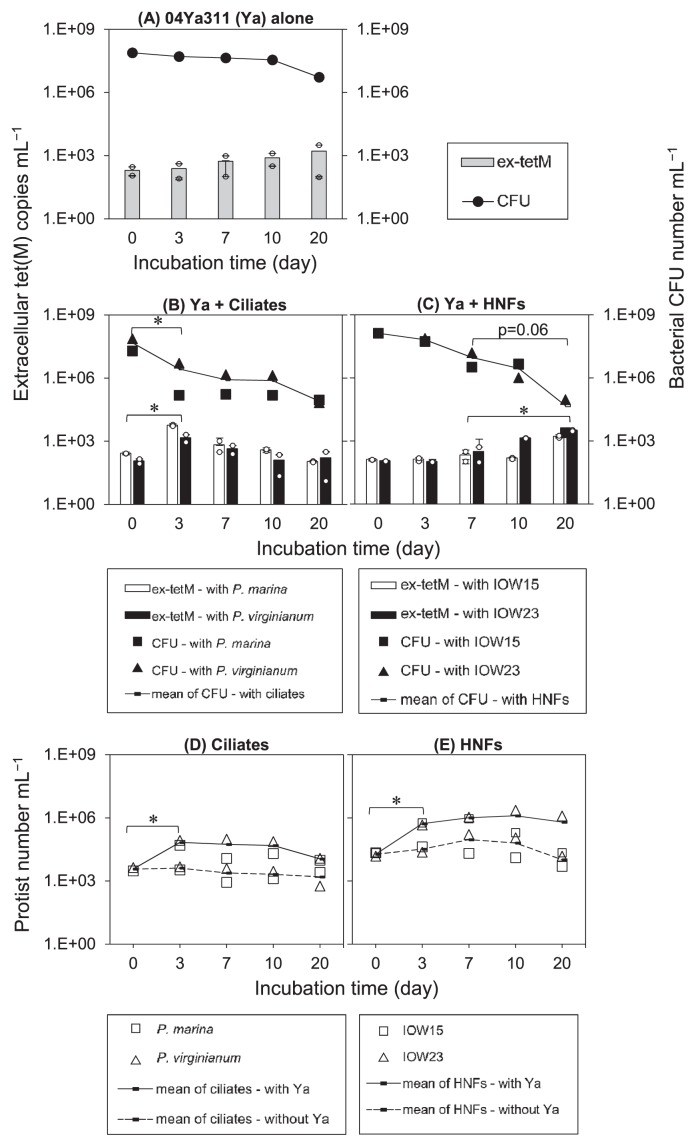
Time-course changes in bacterial densities (lines) and extracellular *tet*(M) copy numbers (bars) (A–C). 04Ya311 alone (A), a co-culture of 04Ya311 with ciliates (B) and with HNFs (C). The protist number in the same microcosms are shown separately in (D, E), where dotted lines indicate the protist alone in control microcosms. Cell numbers (line) are the average of two species, and data from each species are indicated with the symbol placed below the panels. The small circles in bar graphs in A–C show duplicate values of the extracellular *tet*(M) copy number, in which error bars represent the standard error of three measurements of each qPCR value. (*) ex-tetM copies mL^−1^ and bacterial CFU mL^−1^ show significant differences (*p*<0.05) between pairs of time points.

The initial conditions of the microcosms are summarized in Table 1. Time-course changes in the abundances of bacteria and protists in the microcosms are shown in Fig. 1 with line graphs. The inoculated number of 04Ya311 was 1.7×10^7^–1.4×10^8^ CFU mL^−1^. The number of ciliates was 2.1–5.1×10^3^ cells mL^−1^ and HNFs was 6.6×10^3^–2.7×10^4^ cells mL^−1^. Cell densities in the mono-culture microcosms of bacteria (Fig. 1A) and protists (Fig. 1D and E) remained constant with negligible fluctuations. In contrast, in the co-culture microcosms containing bacteria and ciliates, the bacterial number significantly decreased (*p*<0.05) (Fig. 1B), while the ciliate number significantly increased to 4.1–5.1×10^4^ (*P. marina*) and 8.7–9.9×10^4^ cells mL^−1^ (*P. virginianum*) by d 3 (*p*<0.05) (Fig. 1D), and then remained constant throughout the incubation period. In the case of HNFs, bacteria in the IOW15 and IOW23 microcosms decreased from 1.3×10^8^ to 6.5×10^7^ CFU mL^−1^ by d 3 (Fig. 1C). However, HNFs significantly increased to 4.22–6.05×10^5^ cells mL^−1^ (*p*<0.05) on d 3 (Fig. 1E). Slight decreases in bacteria in HNF co-cultures were found from d 7 to 20 (*p*=0.06) (Fig. 1C), and reached 2.5×10^3^ and 9.3×10^4^ CFU mL^−1^ with IOW15 and IOW23, respectively.

Grazing rates in the initial period by d 3 were calculated for ciliates and HNFs, with average values from two strains. In ciliates, bacteria decreased to 7.2×10^1^ CFU (Fig. 1B), whereas ciliates increased to 1.8×10^1^ cells (Fig. 1D). The bacteria/ciliate rate was 4.1. On the other hand, in HNFs, bacteria decreased to 0.2×10^1^ CFU (Fig. 1C), whereas HNFs increased to 1.0×10^1^ cells (Fig. 1E). The bacteria/HNF rate was 0.2. These calculations indicate that ciliates grazed on a 20.5-fold greater number of bacteria than HNFs. A previous study showed that the grazing rate of ciliates was 28-fold greater than that of HNFs (22); therefore, grazing rates in the present experiments by both protists are a likely consequence.

**Fig. 2:**
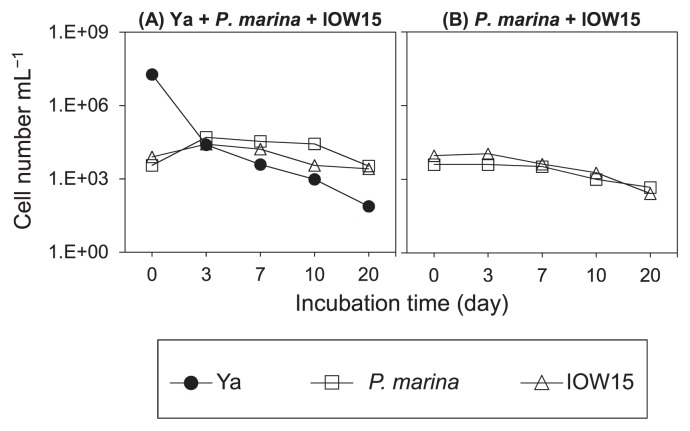
Cell number in a co-culture of three microbes. A bacterium (Ya), ciliate (*P. marina*), and HNF (IOW15) are employed. A mixture of the three species (A), and a mixture of *P. marina* and IOW15 without 04Ya311 (B). Each value is the average of two independent experiments.

In order to provide evidence to clarify interactions between microbial communities in seawater, the tripartite interactions of ciliates, HNFs, and bacteria were also examined by co-culturing *P. marina*, IOW15, and 04Ya311 (Fig. 2). The bacterial number rapidly decreased (approximately 1/1000), whereas the number of ciliates and HNFs slightly increased from 3.5×10^3^ (*P. marina*) and 8.0×10^3^ (IOW15) to 4.4–5.0×10^4^ and 2.6–3.1×10^4^ cells mL^−1^, respectively, by d 3 (Fig. 2A). Bacteria were consumed by grazers during the co-culture. Active cell growth was not observed in any protist microcosm after d 3 (Fig. 1D, 1E, Fig. 2A), and this may be attributed to a spatial effect; however, the underlying mechanisms currently remain unclear. In bacteria-free microcosms (Fig. 2B), the densities of ciliates and HNFs slightly decreased during the culture period.

### Copy number of ex-tetM

ex-tetM was detected during incubations in all microcosms with 04Ya311 (Fig. 1, bar graphs). In the microcosm containing 04Ya311 only, ex-tetM slightly increased from 1×10^2^ copies mL^−1^ to 5.6–8.3×10^2^ copies mL^−1^ by d 20 (Fig. 1A). During grazing stress, a marked increase in ex-tetM was found in the presence of ciliate strains. The copy number on d 0 was 8.72×10^1^–2.7×10^2^ copies mL^−1^, which increased to 9.2×10^2^–6.25×10^3^ copies mL^−1^ by d 3 (*p*<0.05). It then slightly decreased to the initial level (Fig. 1B). Gradual increases in ex-tetM were observed from d 7 (1.02–5.33×10^2^ copies mL^−1^) to d 20 (1.48–3.46×10^3^ copies mL^−1^) (*p*<0.05) in the HNF co-cultures (Fig. 1C). Increases were similar within the two species of ciliates and HNFs.

### Abundance of intracellular *tet*(M) in the community

**Fig. 3:**
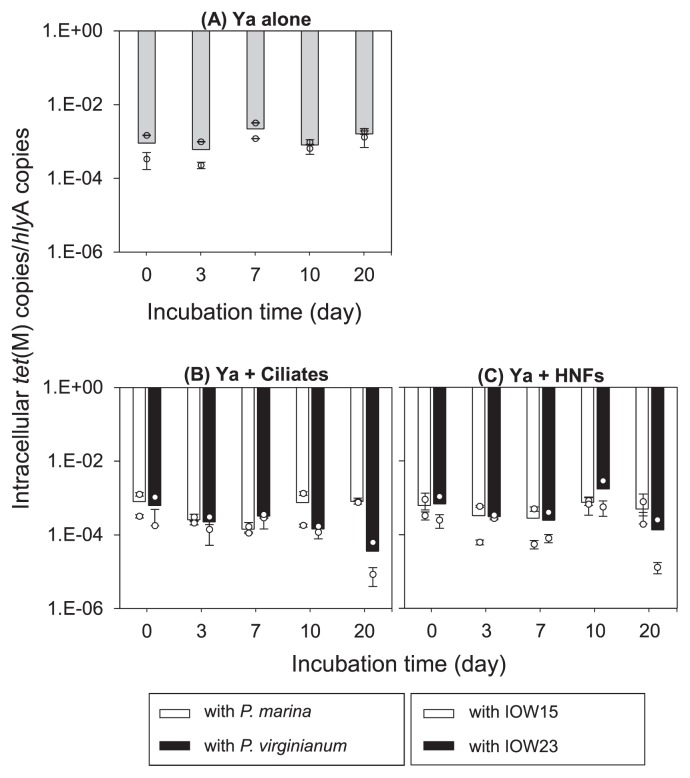
Intracellular *tet*(M) copy number in a population of 04Ya311 alone (A), 04Ya311 with a ciliate (B), and 04Ya311 with a HNF (C). Each bar shows the average of two independent experiments, and the small circles are duplicate values. Error bars represent a standard error of three measurements of each qPCR value.

The abundance of pAQU1 in the 04Ya311 population with ciliates and HNFs was assessed in cellular fractions by measuring the *tet*(M) copy number normalized with *hlyA* (Fig. 3). In cultures of 04Ya311 alone, the *tet*(M) copy number in the bacterial population was nearly constant with minor fluctuations, varying from 6.05×10^−4^ to 2.20×10^−3^ (Fig. 3A). Minor fluctuations (in the range of 1.42–8.14×10^−4^) were observed in the microcosms of co-cultured 04Ya311 and *P. marina*, while microcosms with *P. virginianum* slightly decreased from 6.25×10^−4^ (d 0) to 3.59×10^−5^ (d 20) (Fig. 3B). Minor fluctuations were observed in HNFs, with values ranging from 2.80 to 7.59×10^−4^ in IOW15 and from 1.34×10^−4^ to 1.75×10^−3^ in IOW23 (Fig. 3C). The copy number of pAQU1 was approximately one per cell (1.04 copy cell^−1^, *n*=10), which was calculated from data reported by Bien *et al.* (4). However, the copy number of *hlyA* coded on the 04Ya311 chromosome is currently unknown. Thus, the number of *tet*(M) copies normalized by *hlyA* was calculated to be approximately 10^−3^ (Fig. 3)

## Discussion

A previous study reported that extracellular ARGs are found in water environments ranging from 2.7×10^2^ to 4.58×10^4^ gene copies L^−1^ (54), and are derived from dead ARB (51), the secretion of live ARB (41), or possibly from grazing on ARB by grazers (1, 18, 31). Interactions between bacteria and ciliates/flagellates/algae have also been suggested to release exDNA into freshwater environments (1, 21, 31). However, limited information is available on the effects of marine ciliates and HNFs on the release of ARGs/resistance plasmids from ARB. In most studies, the concentration of exDNA released by grazing was measured with a fluorometric reagent, such as Hoechst (21, 53) or 3,5-diaminobenzoic acid (DABA) (1). These methods measure a mixture of exDNA derived from bacteria and grazers. Our study using qPCR is a suitable method for measuring extracellular ARGs released from ARB under grazing pressure.

In the present study, we aimed to quantitatively show the effects of grazing pressure on ARG formation in the environment. We found that ciliates and HNFs are involved in the release of ARGs under oligotrophic conditions (Fig. 1). ex-tet(M) increased with decreases in bacteria; however, the time course for increasing profiles differed between ciliate and HNFs. ex-tetM markedly increased by d 3 in the ciliate microcosm (Fig. 1B) and by d 20 in the HNF microcosm (Fig. 1C). Regarding the effects of ciliates on exDNA formation, a co-culture of *E. coli* with *T. thermophila* showed that exDNA (0.03 μg mL^−1^ on d 0) increased to 0.24 μg mL^−1^ by d 12 with elevations in ciliates from 10^1^ to more than 10^4^ cells mL^−1^ (21).

We found that time course increases in ex-tetM differed between the ciliate and HNF co-cultures. The difference in ex-tetM production between ciliates and HNFs may have been caused by variations in the grazing rate. The total grazing rate of ciliates in an aquatic environment was previously reported to be 28-fold higher than that of HNFs (22). The present study showed that the grazing rate of ciliates was 20.5-fold higher than that of HNFs. Differences in the increase kinetics of exDNA have been suggested to depend on the initial ratio of grazer and bacteria densities (11, 52) and the utilization of exDNA as a nutrient source by bacteria under oligotrophic conditions (12).

The stabilization of exDNA is suspected to be due to the attachment to particles and metals, which avoids digestion by nucleases (51). If these mechanisms work in an actual water environment, ex-tet(M) may be relatively stable and possibly become a source of horizontal gene transfer in the microbial community in natural seawater.

We previously reported that plasmids were retained in various bacterial hosts in an aquatic environment under very low selective pressure by antibiotics (4). The results of the present study (Fig. 3) showed the constant possession of the plasmid pAQU1 by 04Ya311 cells under grazing pressure. This result was consistent with previous findings by Cairn *et al.* (6), who found that grazing by the ciliate *T. thermophile* increased the prevalence of the conjugative plasmid RP4 in the pathogenic bacterium *Serratia marcescens* with or without kanamycin. Protists keep bacterial growth constant, and, thus, the bacterial conjugative plasmid is not lost due to activation of the conjugation system (6, 24). Hence, the conjugative plasmid may be maintained in the presence of grazers. In the present study, the copy number of *tet*(M) in bacterial cells remained constant, although a slight decrease in *tet*(M) was observed in bacteria with *P. virginianum* (Fig. 3B). The difference between *P. marina* and *P. virginianum* may be due to the variations in grazing features between the species.

In conclusion, we herein identified novel aspects to show that ARGs released by grazing pressure may be preserved in seawater. ARGs on plasmids may continue to be possessed by bacterial cells in the presence of grazers. ex-tet(M) and bacterial *tet*(M) may form the high background of the environmental ARG pool. These results provide a better understanding of the formation of an ARG pool and the dissemination of ARGs in natural seawater.

## Supplementary



## Figures and Tables

**Table 1:** Initial community composition in each microcosm

Microcosm	04Ya311 (CFU mL^−1^)	*P. marina* (cells mL^−1^)	*P. virginianum* (cells mL^−1^)	IOW15 (cells mL^−1^)	IOW23 (cells mL^−1^)
Mono-culture					
04Ya311 alone	6.5–9.1×10^7^	—	—	—	—
*P. marina* alone	—	2.1–4.1×10^3^	—	—	—
*P. virginianum* alone	—	—	3.9–5.1×10^3^	—	—
IOW15 alone	—	—	—	2.3–2.7×10^4^	—
IOW23 alone	—	—	—	—	1.4–1.6×10^4^

Co-culture					
04Ya311+*P. marina*	1.7–2.3×10^7^	2.1–4.1×10^3^	—	—	—
04Ya311+*P. virginianum*	6.2–8.4×10^7^	—	3.9–5.1×10^3^	—	—
04Ya311+IOW15	1.3–1.4×10^8^	—	—	2.3–2.7×10^4^	—
04Ya311+IOW23	1.3–1.4×10^8^	—	—	—	1.4–1.6×10^4^
04Ya311+*P. marina*+IOW15	1.8–1.9×10^7^	3.3–3.6×10^3^	—	6.6–9.3×10^3^	—
