# Supplementary material for: Release and Constancy of an Antibiotic Resistance Gene in Seawater under Grazing Stress by Ciliates and Heterotrophic Nanoflagellates
Source: Microbes Environ. 2017 Jun 8;32(2):174–9. doi: 10.1264/jsme2.ME17042 (PMC5478541; doi:10.1264/jsme2.ME17042)
Supplement: Supplementary file 1 [file 32_174_s1.pdf]

Supplementary

A

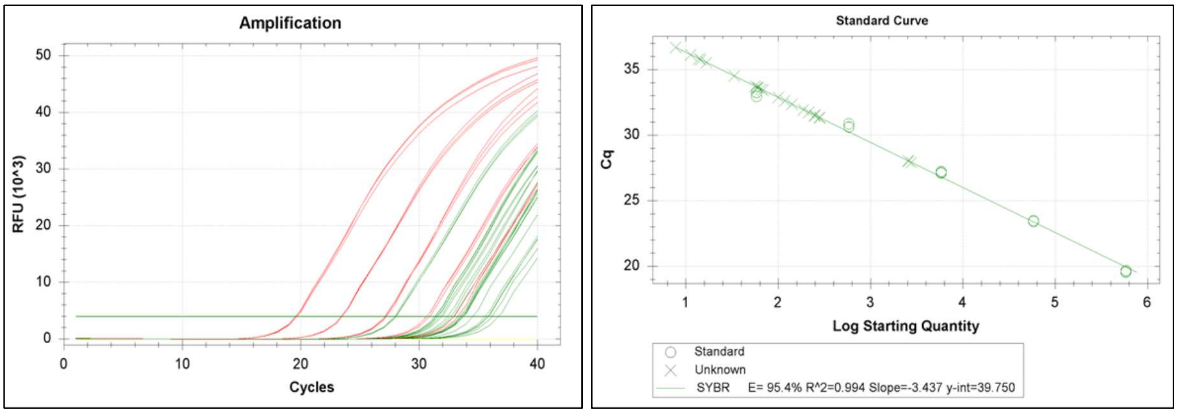

B

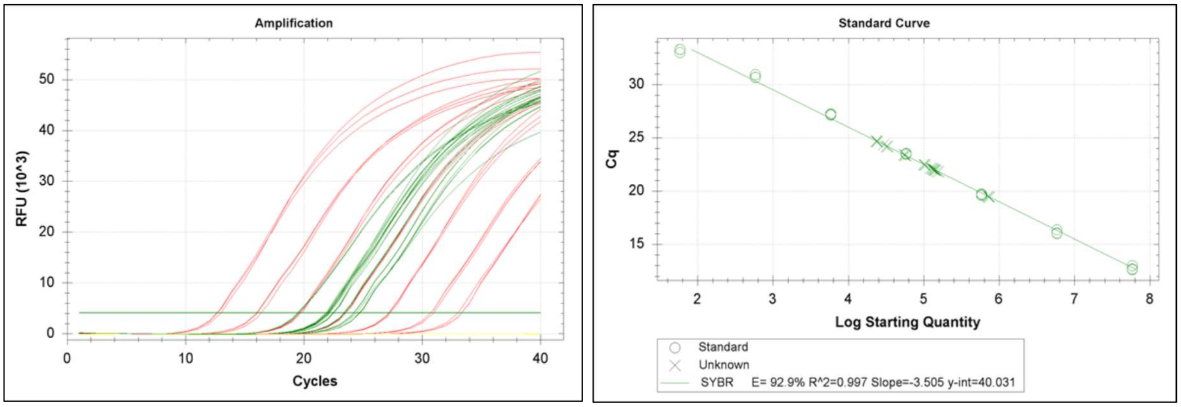

C

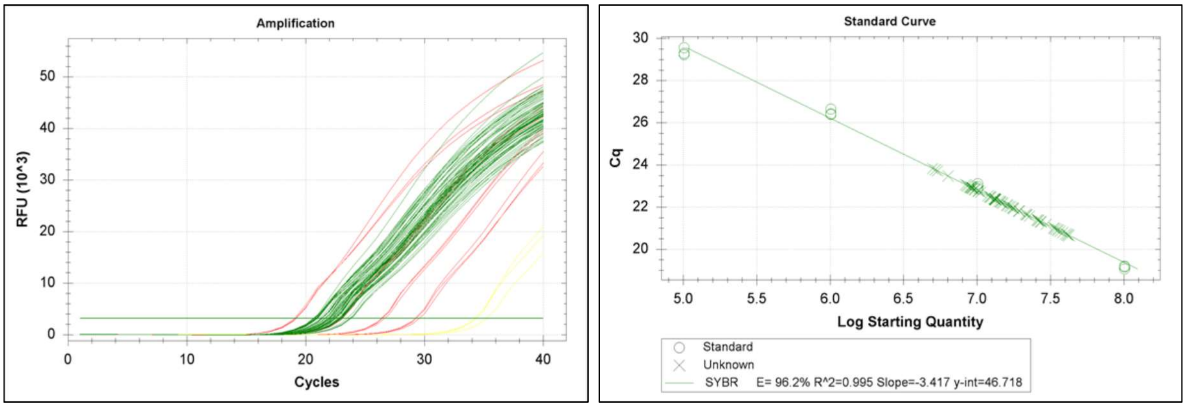

Figure S1 qPCR standard curve for measurement of **(A)** extracellular *tet*(M), amplification efficiency = 95.4%, linearity (R^2) = 0.994; **(B)** intracellular *tet*(M), amplification efficiency = 92.9%, linearity (R^2) = 0.997; and **(C)** *hly*A, amplification efficiency = 96.2%, linearity (R^2) = 0.995. *RFU= Relative Fluorescence Unit.*
